# Alleviation of Copper-Induced Stress in Pea (*Pisum sativum* L.) through Foliar Application of Gibberellic Acid

**DOI:** 10.3390/biology10020120

**Published:** 2021-02-05

**Authors:** Talha Javed, Muhammad Moaaz Ali, Rubab Shabbir, Raheel Anwar, Irfan Afzal, Rosario Paolo Mauro

**Affiliations:** 1 College of Agriculture, Fujian Agriculture and Forestry University, Fuzhou 350002, China; talhajaved54321@gmail.com (T.J.); rubabshabbir28@gmail.com (R.S.); 2 Seed Physiology Lab, Department of Agronomy, University of Agriculture, Faisalabad 38040, Pakistan; irfanuaf@gmail.com; 3 College of Horticulture, Fujian Agriculture and Forestry University, Fuzhou 350002, China; muhammadmoaazali@yahoo.com; 4 Institute of Horticulture Sciences, University of Agriculture, Faisalabad 38040, Pakistan; raheelanwar@uaf.edu.pk; 5 Dipartimento di Agricoltura, Alimentazione e Ambiente (Di3A), Università degli Studi di Catania, Via Valdisavoia, 5-95123 Catania, Italy

**Keywords:** *Pisum sativum* L., copper and oxidative stresses, gibberellic acid, foliar application

## Abstract

**Simple Summary:**

Phytohormones are key regulators of several stages of plant growth and development as well as provide the regulatory response against various heavy metals stresses by mediating physio-morphological responses and enzymatic activities. The current study evaluated the effects of gibberellic acid (GA_3_) foliar applications on the performance of pea grown either in Cu-contaminated (Cu+) and non-contaminated (Cu−) soil. GA_3_ was applied exogenously (0, 10, 50, and 100 mg·L^−1^) on 15-days-old plants, and the results show that the increasing concentration of GA_3_ buffered the phytotoxic effects of Cu, coupled with an increase in plant growth and physiological variables. The results also showed that foliar-applied GA_3_ up to 100 mg·L^−1^ alleviated the oxidative stress, as inferred from the lower concentrations of malondialdehyde (MDA) and H_2_O_2_, which mirrored the increased activity of antioxidant enzymes, i.e., superoxide dismutase, peroxidase, and catalase. In addition, enhanced growth, physiology, and enzymatic activities were also observed in pea plants sprayed with GA_3_ up to 100 mg·L^−1^ in Cu− soil. Overall, the foliar application of GA_3_ boosted phytoextraction of Cu from the soil and alleviated the oxidative stress in pea plants grown in Cu-polluted soil.

**Abstract:**

Copper (Cu) is an essential metal for plants. However, its excess in soil can adversely affect plant metabolism. The current study evaluated the effects of gibberellic acid (GA_3_) foliar applications on the performance of pea plants grown either in Cu-contaminated (Cu+) and non-contaminated (Cu−) soil. GA_3_ was sprayed (0, 10, 50, and 100 mg·L^−1^) on 15-days-old plants. The results showed that the increasing concentration of GA_3_ buffered the phytotoxic effects of Cu and enhanced plant growth, photosynthesis, and leaf chlorophyll content. Foliar-sprayed GA_3_ up to 100 mg·L^−1^ alleviated the oxidative stress, as inferred from the lower concentrations of MDA and H_2_O_2_ (33.3 µmol·g^−1^ and 182 µmol·g^−1^, respectively), and boosted the activity of superoxide dismutase (64.4 U·g^−1^·FW), peroxidase (122.7 U·g^−1^·FW), and catalase (226.3 U·g^−1^·FW). Interestingly, GA_3_ promoted Cu accumulation in different plant parts when compared to untreated plants, likely due to increased photosynthetic and transpiration rates. Overall, foliar application of GA_3_ promoted phytoextraction of Cu and alleviated the oxidative stress in pea plants grown in Cu+ soil.

## 1. Introduction

Pea (*Pisum sativum* L.) is an important leguminous crop, whose high protein content (up to 22–23%) is almost three times that of cereals [1]. The crop is mainly cultivated in tropical and sub-tropical regions of the world, and has the ability of nitrogen (N) fixation in soil through N fixing bacteria residing in roots. It has a deep root system, and hence is highly drought tolerant [2]. It has good nutritive value, as it contains considerable amounts of lysine, thiamine, riboflavin, iron, and niacin, and its product is represented by green pods or mature seeds as meal worldwide [2]. In pea, physio-morphological and biochemical events are the most crucial on which the whole productivity relies, and both are influenced by metals toxicity [3]. Heavy metals contamination is becoming increasingly common in many agricultural farmlands worldwide, due to long-term mining activities which endanger crops yield and food security [4,5]. This feature is of major concern in agricultural systems, due to the adverse effects on crop growth and physiology (phytotoxicity), food safety and marketability, soil micro biota, and most importantly on human health [6]. In addition, the dynamic equilibrium between production and elimination of reactive oxygen species (ROS) under normal growth conditions can be altered due to toxic levels of heavy metal, which results in disruption of structure and functioning of cell membranes as a result of lipid peroxidation, malondialdehyde (MDA), and hydrogen peroxide (H_2_O_2_) accumulation [7]. Nevertheless, remediation of metal-contaminated soils by conventional (physical and chemical) methods is not ideal, as it requires huge resources, is time consuming, and sometimes even environmentally hazardous [8]. However, more recent emerging technologies, such as exogenous application of phyto-hormones, due to their ease, efficiency, and environmentally friendliness for phytoremediation should be considered for remediation [4]. 

Gibberellic acid (GA_3_) is a plant hormone involved in several stages such as cell division, plant height, tissue differentiation, dry matter accumulation, net assimilation rate, leaf expansion, elongation, flowering, photosynthesis, and transpiration rate [9,10,11]. In addition, GA_3_ is a kind of diterpenoid compound, and is known to play key roles to enhance phytoremediation efficiency of many crops by mediating physiology, morphology, and enzymatic activities [12,13]. Previous literature reported that exogenous supplementation of GA_3_ as foliar spray significantly enhanced growth and phytoextraction efficiency of different crop plants such as *Zea mays* L., *Tagetes patula* L., *Solanum nigrum* L., and *Corchorus capsularis* L. grown on Cu, cadmium (Cd), lead (Pb), and benzo[a]pyrene-contaminated soil [4,13,14,15]. 

Exogenous applications of GA_3_ have a remarkable role in *S. nigrum* for substantial increases in growth and development [13]. In addition, a considerable increase in growth and biomass accumulation of *Carapichea ipecauanha* was observed in plants that received foliar applied GA_3_ as compared to untreated plants [16]. Previous literature also reported the protective role of GA_3_ with improved photosynthetic performances in chromium (Cr)-contaminated soil [10]. The possible reason behind this mechanism might be the enhanced antioxidant activities that reduced the oxidative damage of *Corchorus capsularis* L. plants growing in Cu-contaminated soil [4]. There is a lot of literature on exogenous supplementation of GA_3_ to enhance plant growth and development in metal-contaminated soil, however, available on other plant species, such as and *Solanum nigrum* L., and *Tagetes patula* L. [12,15]. The uniqueness of *Pisum sativum* L. plants due to high nutritive value and negative impacts of Cu on human health demands phytoremediation; however, sufficient information is not available regarding Cu tolerance, antioxidative defense mechanism, and Cu accumulation in different parts of this species, when grown under different concentrations of foliar applied GA_3_. Therefore, it is vital to explore the protective and growth promoting role of GA_3_ in Cu-contaminated soils. It may be hypothesized that GA_3_ alleviates Cu and oxidative stresses by modulating some physio-morphological and biochemical processes in pea. To test this hypothesis, pea plants were germinated on Cu-contaminated soil and then foliar sprayed with 10, 50, and 100 mg·L^−1^ GA_3_ at 15 days after germination. In addition to evaluating key stress benchmarks on plant growth (plant height, plant weight, leaf chlorophyll, photosynthesis, and transpiration rates), accumulation of electrolytes, H_2_O_2_ and MDA, and activities of key of radical oxygen-scavenging enzymes catalase, peroxidase, and superoxide dismutase were also quantified. 

## 2. Materials and Methods 

### 2.1. Experimental Site and Growth Conditions 

The soil for the pot experiment was collected from the Research Area, Department of Agronomy, University of Agriculture, Faisalabad City, Punjab Province, Pakistan (30.37° N, 69.34° E) at depth of 0–20 cm. After grinding, the soil was mixed thoroughly, air dried under shade, and sieved through a 5 mm sieve before filling in pots (10 kg soil per pot). The textural class of soil for experimentation was loam and, before the start of the experiment, had the following characteristics: Electrical conductivity (EC) 0.432 dSm^−1^, pH 7.5, available K 344 mg·kg^−1^, available P 12.21 mg·kg^−1^, and Cu 109 mg·kg^−1^. Total Cu (2000 mg·kg^−1^), as CuSO_4_·5H_2_O, was mixed thoroughly in soil prior to filling in pots, to simulate Cu contamination (Cu+ soil) [4]. The same number of experimental units without Cu addition in the soil were included as control (Cu− soil). A pea variety with indeterminate growth habit, “Sarsabz” (*Pisum sativum* L.) with 98% germination capacity was procured from Ayub Agricultural Research Institute, Faisalabad, Pakistan and used throughout the course of the experiment. The experiment was conducted in the glasshouse at the Research Area, Department of Agronomy, University of Agriculture, Faisalabad, from November 2018 to January 2019. Plants in the glasshouse received natural light, with day/night average temperature of 26/15 °C and day/night average humidity of 65/80%. The bi-factorial experiment was laid out under completely randomized design (CRD), with four replications (each replication contained five pots for each treatment). Ten seeds were directly sown in each pot. Each replication contained 50 plants of each experimental unit. Irrigation, weeding, and necessary agronomic practices, based on physical observations, were done when needed.

### 2.2. Exogenous Application of GA_3_

Foliar sprays of GA_3_ were done 14 days after seeding. From 10:00 until 11:00 am, treatments, i.e., 0, 10, 50, 100 mg·L^−1^ were applied by exogenously spraying GA_3_ on whole seedlings until solution falls and treatments were applied only once during the whole experiment [4].

### 2.3. Plant Growth Attributes

Five uniform plants were randomly selected from each replication to determine the number of leaves, plant height, and plant relative fresh and dry weights at 40 days after seeding (DAS). To determine relative fresh and dry weights, plants were washed with distilled water, dried with paper and weighed for their fresh weight followed by oven drying at 70 °C until constant weight achieved. After oven drying, dry weights were recorded.

### 2.4. Physiological Variables

At 40 DAS from five uniform randomly selected plants from each replication, leaf chlorophyll content was measured with a chlorophyll SPAD meter (CCM-200 plus, Opti-Sciences, Hudson, NH, USA) according to manufacturer’s instructions, and presented as SPAD values. On the same date measurements of net photosynthetic rate (µmol CO_2_ m^−2^·s^−1^), stomatal transpiration rate (mmol H_2_O m^−2^·s^−1^), and CO_2_ concentration (µmol·mol^−1^) were made on fully expanded leaves from the top of the plant canopy by using an open system LCA-4 (ADC BioScientific Ltd., Hoddesdon, UK). These measurements were made from 10:00 to 11.00 a.m., with the following specifications: Ambient pressure (P) 88.76 kPa, molar flow of air per unit leaf area (Us) 201.89 mol m^−2^·S^−1^, temperature of leaf chamber (Tch) varied from 38.9 to 42.4 °C, leaf chamber molar gas flow rate (U) 236 µmol·S^−1^, leaf surface area 10.11 cm^2^, and photosynthetically active radiation (PAR) at leaf surface was maximum up to 887 µmol m^−2^·s^−1^.

### 2.5. Oxidative Stress Indicators and Antioxidant Response

To determine malondialdehyde (MDA) content, an indicator of lipid peroxidation, 0.1 g leaves were ground with 25 mL of 50 mM phosphate buffer solution containing 1% polyethylene pyrrole with the help of pestle and mortar. After centrifugation at 12,000× *g* for 15 min at 4 °C, the supernatant was taken, followed by heating at 100 °C for 20 min. The tubes were quickly cooled in an ice bath after heating. The absorbance was taken at wavelengths of 532, 600, and 450 nm by using a spectrophotometer (T60 U Spectrophotometer, PG Instruments Ltd., Leicestershire, UK) [17]. 

To determine H_2_O_2_ concentration, leaf samples (1 g) were ground in 9 mL of normal saline solution (4.5 g NaCl added in 500 mL ddH2O) followed by centrifugation at 10,000× *g* for 10 min. Three tube types were prepared, namely empty, standard, and sample tubes. Briefly, reagent 1 and 2 (1.0 mL) in all tubes, H_2_O (0.1 mL) in empty tube, standard solution (0.1 mL) in standard tube, and sample (0.1 mL) in sample tube was added. The absorbance was taken at 405 nm with spectrophotometer according to H_2_O_2_ determination kit (Nanjing Jiancheng Biology Co., Ltd., Nanjing, China).

To determine electrolyte leakage (EL), fully expanded leaves from the top of the plant canopy were taken, followed by cutting into minor slices (5–6 mm length), placed in sterilized test tubes having 8 mL distilled water, incubated, and transferred to water bath for 12 h prior to measuring the initial electrical conductivity (EC_1_). After measuring the initial EC_1_, samples were autoclaved at 121 °C for 20 min followed by cooling down to 25 °C to measure the final electrical conductivity (EC_2_). To measure the electrolyte leakage, a pH/conductivity meter (INCO-LAB Company, Al Kuwayt, Kuwait) was used, then the following equation for EL calculation was applied:

EL = (EC1/EC2) × 100

To determine antioxidant activities, 0.5 g leaves were ground using a tissue grinder in 8 mL of cooled phosphate buffer (pH 7.0, containing 1% (w/v) polyvinylpyrolidone) in test tubes. The homogenate was centrifuged at 15,000 rpm for 20 min at 4 °C. The supernatant was used for assays of enzymes activity. The activity of catalase (CAT) and peroxidase (POD) was measured by using the method of Maehly [18]. The reaction solution (3 mL) contained 0.1 mL standard enzyme extract, 15 mM H_2_O_2,_ and 50 mM phosphate buffer (pH 7.0). The absorbance was taken at 240 nm with the spectrophotometer. The POD reaction solution (3 mL) contained 0.1 mL enzyme extract, 50 mM sodium acetate buffer (pH 5.0), 40 mM H_2_O_2_, and 20 mM guaiacol. The absorbance was taken at 470 nm. The superoxide dismutase (SOD) reaction solution (3 mL) contained 1.3 µM riboflavin, 50 µL enzyme extract, 50 µM nitroblue tetrazolium (NBT dissolved in ethanol), 13 mM methionine, 50 mM phosphate buffer (pH 7.8), and 75 nM EDTA [19]. The absorbance was taken at 240 nm.

### 2.6. Copper Concentration in Roots, Leaves and Stems

To determine the Cu content, in root, leaves, and stems, respective parts from ten uniform randomly selected plants were taken, oven dried, and ground at 40 DAS. Briefly, 0.1 g of the respective ground sample was digested in HNO_3_/HClO4 (4:1) solution followed by dilution of digested sample in de-ionized water up to final volume of 25 ml. The supernatant was taken and passed through a filter paper. Copper standard solution (SRM-3114, 10% HNO_3_, Sigma-Aldrich, Milwaukee, WI, USA) was used as a primary calibration standard for the quantitative determination of copper in roots, leaves, and stem of pea. Readings were taken by using a Perkin-Elmer 3100 Atomic Absorption Spectrophotometer (Thermo Fisher Scientific, Lancashire, UK).

### 2.7. Statistical Analysis

A two-way analysis of variance (Cu contamination of soil × GA_3_ application) was executed to evaluate the effects of GA_3_ on the recorded variables both under normal (without Cu stress, control) and under Cu-contaminated soil conditions. Tukey’s honestly significance difference (HSD) test was used for comparison of treatment means (*p* ≤ 0.05), using a statistical software package ‘Statistix 8.1’ (https://www.statistix.com/). A principal component analysis among treatments and dependent variable was executed using XLSTAT ver. 2018 (https://www.xlstat.com/) to delineate the effect of different doses of GA_3_ on physiological growth attributes of pea plants grown in Cu− and Cu+ soil. Clustering of observations and variables into groups were done on the basis of their highest squared cosine values corresponding to factors, F1 and F2. Correlation coefficients among variables were determined with Pearson (*n*) method.

## 3. Results

### 3.1. Plant Growth Attributes

The results revealed that the untreated plants grown in Cu+ soil exhibited the minimum plant height (19.33 cm) as compared to the other treatments. Though all foliar applied GA_3_ concentrations enhanced the plant height of pea, however, the plants treated with 100 mg·L^−1^ GA_3_ showed the maximum height in both soils (40.1 cm in Cu− soil; 34.2 cm in Cu+ soil). Similarly, maximum plant relative fresh weight (34 g) was recorded in plants receiving 100 mg·L^−1^ GA_3_ grown in Cu− soil followed by the plants receiving same treatment grown in Cu+ soil, indicating that GA_3_ increased the above ground biomass accumulation in pea (Table 1).

In the case of plant dry weight, the plants treated with 100 mg·L^−1^ GA_3_ showed the highest values in both soils (17.2 g Cu− soil and 15.2 g Cu+ soil) followed by 50 and 10 mg·L^−1^ GA_3_ (Table 1). Similar to the aforesaid variables, plants treated with GA_3_ showed an increased number of leaves in a dose-dependent manner as compared to control. The plants treated with 100 mg·L^−1^ showed the maximum number of leaves in both soils (30.1 Cu− soil and 28.3 Cu+ soil). The lowest values for plant height, fresh/dry weight, and number of leaves per plant were recorded in control followed by 10 mg·L^−1^ GA_3_ application in both Cu− and Cu+ soil (Table 1).

### 3.2. Physiological Variables

**Figure 1:**
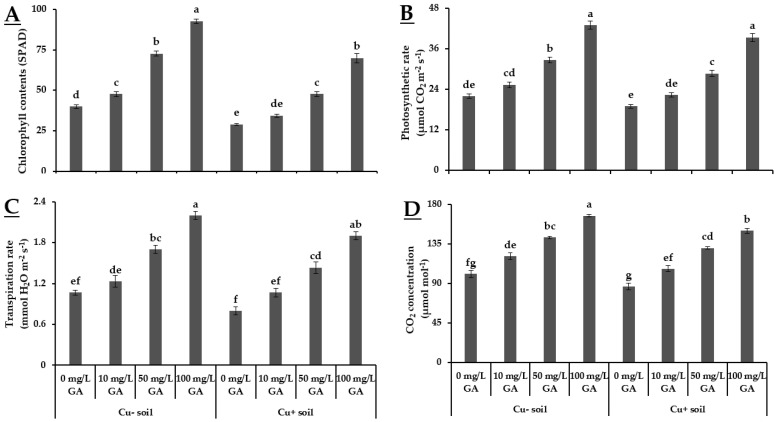
Effect of GA_3_ application on physiological variables of pea grown in Cu+ and Cu− soil. (**A**) Chlorophyll contents; (**B**) Photosynthetic rate; (**C**) Transpiration rate; (**D**) CO_2_ concentration. Different letters indicate significant difference among treatment means according to Tukey’s honestly significant difference (HSD) test (*p* ≤ 0.05). Vertical bars indicate mean ± standard error (*n* = 4, 5 plants per replicate under bi-factorial CRD arrangement).

The results shown in Figure 1 indicate that the plants grown in Cu− soil exhibited better physiological attributes as compared to those grown in Cu+ soil. The exogenous application of GA_3_ improved the performance of pea in terms of their physiological attributes. Specifically, the plants treated with 100 mg·L^−1^ GA_3_ showed the highest chlorophyll content, photosynthetic rate, transpiration rate, and CO_2_ concentration (92.7 SPAD, 43 µmol CO_2_ m^−2^·s^−1^, 2.2 mmol H_2_O m^−2^·s^−1^, and 167.1 µmol·mol^−1^, respectively) followed by plants treated with 50 mg·L^−1^ GA_3_ (72.7 SPAD, 32.6 µmol CO_2_ m^−2^·s^−1^, 1.7 mmol H_2_O m^−2^·s^−1^, and 142.3 µmol·mol^−1^, respectively) and 10 mg·L^−1^ GA_3_ (47.5 SPAD, 25.3 µmol CO_2_ m^−2^·s^−1^, 1.2 mmol H_2_O m^−2^·s^−1^, and 121 µmol·mol^−1^, respectively) in Cu− soil. Similarly, the experimental units received foliar applied 100 mg·L^−1^ GA_3_ showed the highest chlorophyll contents, photosynthetic rate, transpiration rate, and CO_2_ concentration (70 SPAD, 39.3 µmol CO_2_ m^−2^·s^−1^, 1.9 mmol H_2_O m^−2^·s^−1^, and 150 µmol·mol^−1^, respectively) in Cu+ soil. Whereas the untreated plants grown in Cu+ soil showed minimum values of chlorophyll contents, photosynthetic rate, transpiration rate, and CO_2_ concentration (29 SPAD, 19 µmol CO_2_ m^−2^·s^−1^, 0.8 mmol H_2_O m^−2^·s^−1^ and 86.63 µmol·mol^−1^, respectively), showed the positive role of GA_3_ in improving physiological response of pea (Figure 1).

### 3.3. Oxidative Stress Indicators and Antioxidant Response

**Figure 2:**
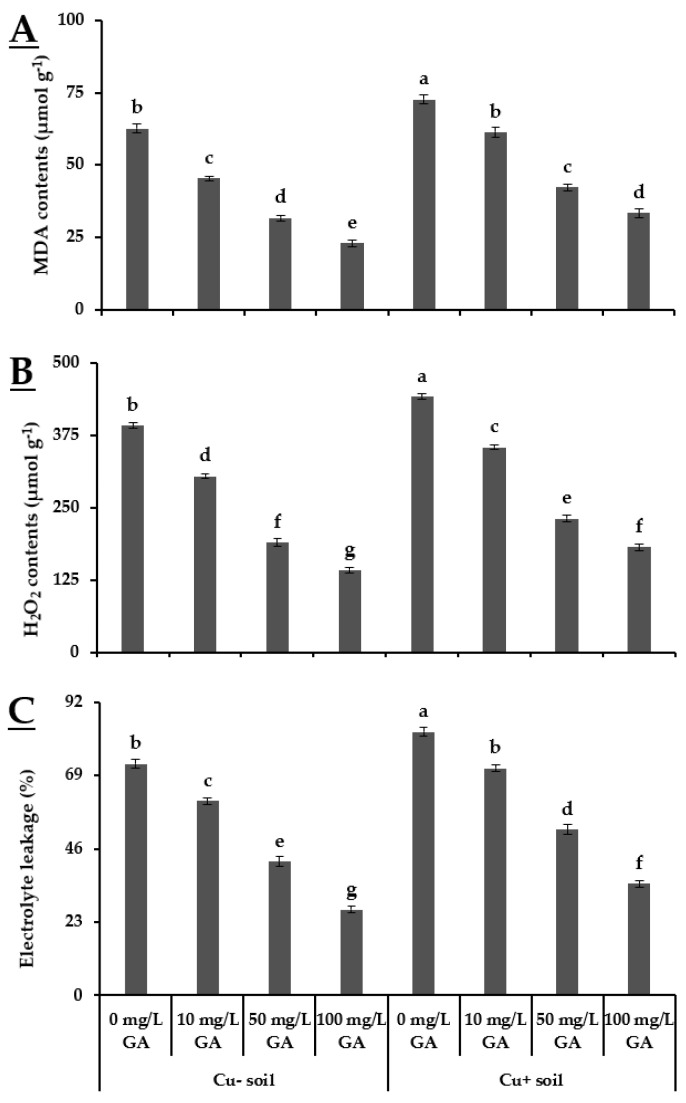
Effect of GA_3_ application on malondialdehyde (MDA), H_2_O_2_, and electrolyte leakage of leaves of pea grown in Cu+ and Cu− soils. (**A**) Malondialdehyde contents; (**B**) H_2_O_2_ contents; (**C**) Electrolyte leakage. Different letters indicate significant difference among treatment means according to Tukey’s honestly significant difference (HSD) test (*p* ≤ 0.05). Vertical bars indicate mean ± standard error (*n* = 4, 5 plants per replicate under bi-factorial CRD arrangement).

Plants grown in Cu− soil, when treated with 100 mg·L^−1^ GA_3_, showed minimum MDA and H_2_O_2_ contents and electrolyte leakage (23 µmol·g^−1^, 143.32 µmol·g^−1^, and 27.2%, respectively). The plants grown in Cu+ soil exhibited increased electrolyte leakage, MDA, and H_2_O_2_ contents than those were grown in Cu− soil. The exogenous application of GA_3_ significantly reduced electrolyte leakage, MDA, and H_2_O_2_ contents in a concentration-dependent manner. The maximum decrease in MDA, H_2_O_2_, and electrolyte leakage were observed in plants treated with 100 mg·L^−1^ GA_3_ in Cu− (63.29%, 63.40%, and 62.84%, respectively) and Cu+ soils (54.12%, 58.79%, and 57.66%, respectively) as compared to other experimental units and control (Figure 2).

**Figure 3:**
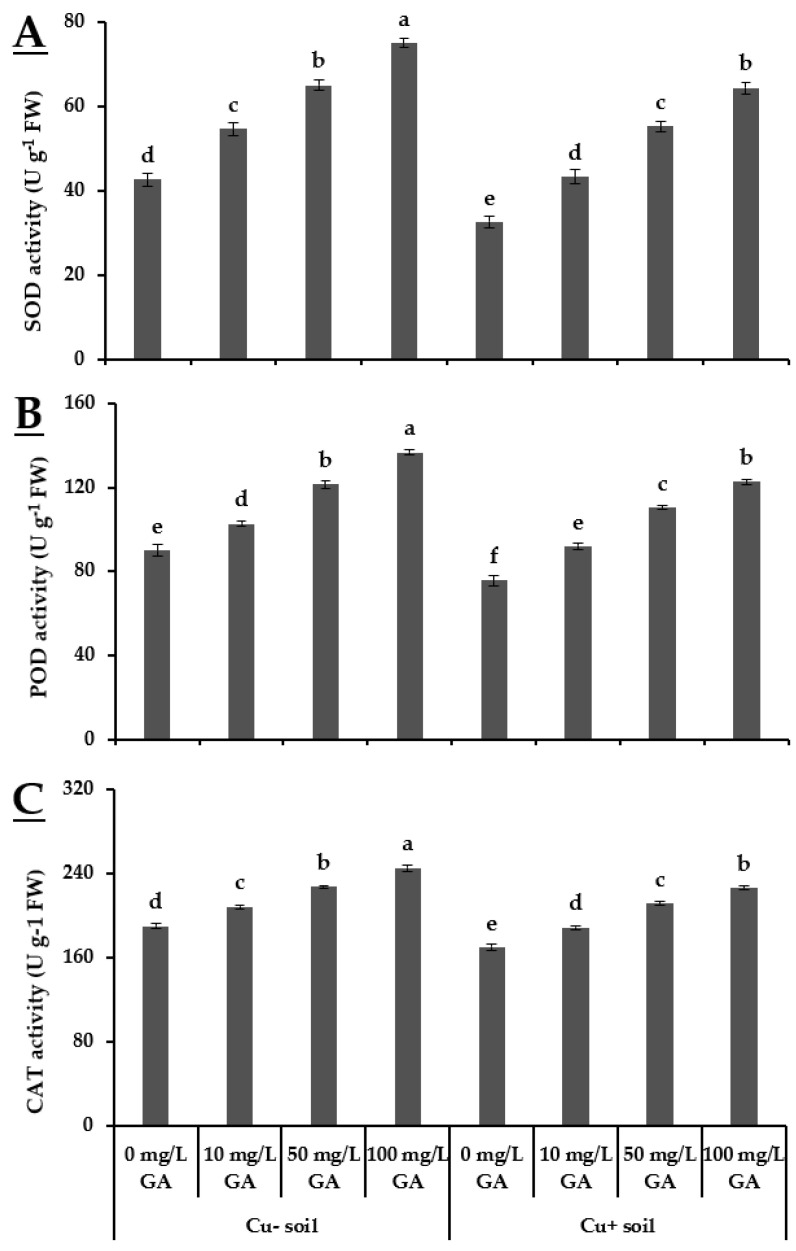
Effect of GA_3_ on activity of reactive oxygen species (superoxide dismutase (SOD), peroxidase (POD), catalase (CAT)) of peas grown in Cu+ and Cu− soils. (**A**) SOD activity; (**B**) POD activity; (**C**) CAT activity. Different letters indicate significant difference among treatment means according to Tukey’s honestly significant difference (HSD) test (*p* ≤ 0.05). Vertical bars indicate mean ± standard error (*n* = 4, 5 plants per replicate under bi-factorial CRD arrangement).

The exogenous application of 100 mg·L^−1^ GA_3_ exhibited maximum SOD activity in the plants grown in Cu− soil (75 U·g^−1^·FW) followed by the plants grown in Cu+ soil (64.39 U·g^−1^·FW). In similarity with the aforementioned variable, the highest POD activity (136.21 U·g^−1^·FW) was also observed in pea plants grown in Cu− soil treated with 100 mg·L^−1^ GA_3_. Plants receiving foliar application of 50 mg·L^−1^ GA_3_ also showed better performance in both soil conditions. In the case of CAT activity, maximum value (245 U·g^−1^·FW) was also recorded in plants treated with 100 mg·L^−1^ GA_3_ in Cu− soil. The reduced activity of antioxidants i.e., SOD, POD, and CAT in untreated plants grown in Cu+ soil indicates a significant effect of Cu stress on pea plants (Figure 3).

### 3.4. Cu Concentration in Roots, Leaves and Stem

The GA_3_-untreated plants grown in Cu- soil exhibited minimum Cu concentration in roots, leaves, and stem of pea plants (16.66, 31.66 and 22.12 mg·kg^−1^, respectively) as compared to all other treatments. The foliar application of GA_3_ enhanced the uptake of Cu in plants. The plants grown in Cu+ soil showed more Cu concentration in roots, leaves, and stem as compared to those grown in Cu− soil. The plants treated with 100 mg·L^−1^ GA_3_ showed maximum Cu concentration in roots, leaves, and stem of peas grown in both soils (34.66, 58.05 and 39.66 mg·kg^−1^ ‘Cu− soil’; 90.66, 193.31 and 145.26 mg·kg^−1^ ‘Cu+ soil’, respectively) (Table 2). 

### 3.5. Multivariate Analysis

**Figure 4:**
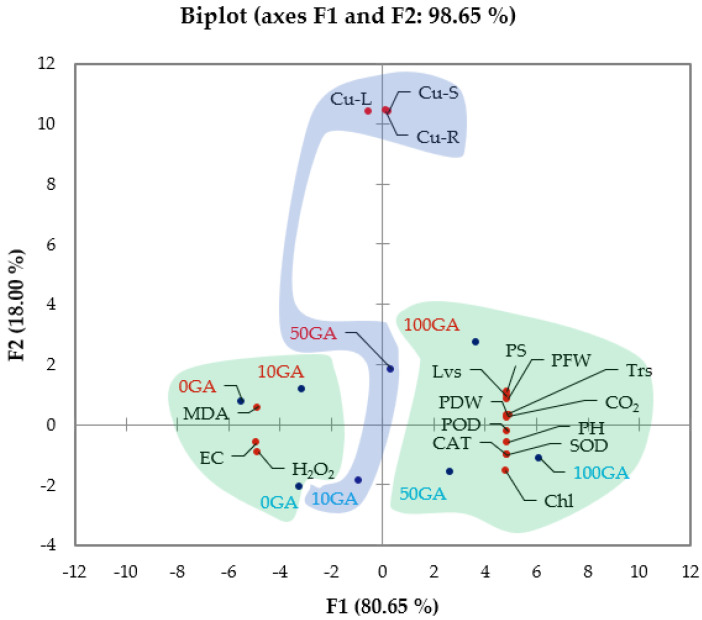
Principal component analysis (biplot) among observations (treatments) and variables (morphological, physiological, and oxidative attributes) of peas. Blue and red colored text depicts GA_3_ treatments on plants grown in Cu− and Cu+, respectively. Clustering of observations and variables into groups (colored shading) is based on their highest squared cosine values corresponding to the factor, F1 (green) and F2 (blue). Abbreviations: 0GA—no GA_3_ treatment; 10GA—10 mg·L^−1^ GA_3_; 50GA—50 mg·L^−1^ GA_3_; 100GA—100 mg·L^−1^ GA_3_; PH—Plant Height; PFW—Plant Relative Fresh Weight; PDW—Plant Dry Weight; Lvs—Number of Leaves; Chl—Chlorophyll Content; PS—Photosynthetic Rate; Trs—Transpiration Rate; CO_2_—CO_2_ Index; MDA—Malondialdehyde Content; H_2_O_2_—H_2_O_2_ content; EC—Electrolyte Leakage; SOD—Superoxide Dismutase; —Peroxidase; CAT—Catalase; Cu-R—Root Cu Content; Cu-L—Leaf Cu Content; Cu-S—Stem Cu Content.

Factor F1, having eigenvalue 13.711 and variability of 80.65% showed positive correlation among plant height, biomass accumulation, photosynthetic rate, transpiration rate, CO_2_ index, H_2_O_2_ contents, SOD, POD, and CAT activity with 50 and 100 mg·L^−1^ GA_3_ treatment in plant grown in Cu− soil, as well as 100 mg·L^−1^ GA_3_ treatment in plant grown in Cu+ soil. Malondialdehyde, electrolyte leakage, and H_2_O_2_ contents are shown in the negative side of the F1 axis, indicating their negative correlation with 100 mg·L^−1^ GA_3_ and positive correlation with control (no GA_3_ treatment) (Table 3) (Figure 4). 

Second factor F2, having eigenvalue 3.059 and variability of 17.99% represented Cu contents in leaves, roots, and stems. Cu contents in leaves, roots, and stem are shown in the positive half of F2 depict their positive correlation with 50 mg·L^−1^ GA_3_ treatment on plants grown in Cu+ soil, while negative correlation with 10 mg·L^−1^ GA_3_ treatment on plants grown in Cu− soil. Thus, principal component analysis delineated the morphological, physiological, and oxidative response of pea plants under the influence of GA_3_ and Cu toxicity.

## 4. Discussion

Several studies have shown that Cu toxicity severely influences growth and productivity of leguminous crops [20,21,22]. In the present study, the pea plants grown in Cu+ soil showed reduced growth and biomass accumulation as compared to the plants grown in Cu− soil (Table 1). Our results are consistent with those of Massoud et al. [23], who reported that the Cu toxicity reduced the fresh biomass of pea plants. The exogenous application of phytohormones is considered a way to mitigate the toxic effects of heavy metals and increase plant tolerance to some abiotic stressors [24]. In our experiment, the foliar applied GA_3_ alleviated the toxic effect of Cu and enhanced plant height, number of leaves, and biomass accumulation in pea plants grown in both Cu+ and Cu− soil (Table 1). These findings are in line with other studies that reported the remarkable role of exogenous application of GA_3_ to enhance plant growth and tolerance to heavy metals such as Cd [25,26,27]. In accordance with our study, the exogenous application of 100 mg·L^−1^ GA_3_ enhanced shoot fresh and dry weight of jute under Cu stress [4]. In the present study, enhanced plant growth variables with the exogenous application of GA_3_ in Cu-stressed plants might be due to better gaseous exchange attributes [4], or GA3 helps in decreasing free metal ions in plants as suggested by Shafigh et al. [28]. 

Gibberellins stimulate plant growth and alleviate the inhibitory effects of different abiotic stressors on plant physiological and growth attributes, such as plant biomass accumulation, chlorophyll, minerals accumulation, gas exchange, electrolyte leakage, as well as the activity of reactive oxygen species [29,30,31]. According to Lüttge [32], photosynthetic efficiency of plants depends on chlorophyll content, which plays a key role in light dependent reaction of photosynthesis. Previous literature also stated that increased production of antioxidants in chloroplast resulted in scavenging of ROS and curtail oxidative damage to photosynthetic membranes [4]. Our findings revealed that chlorophyll content and gas exchanges were significantly influenced by the exogenous application of GA_3_ (Figure 1). Though all GA_3_ concentrations applied improved the physiological attributes in both Cu+ and Cu− soil, higher values were observed in experimental units received 100 mg·L^−1^ GA_3_. The chlorophyll content and gaseous exchange attributes were reduced in Cu+ soil as compared to Cu− soil (Figure 1). The reason behind this phenomenon might be the structural damage of chloroplast resulted from Cu exposure in the soil system [33]. Structural damage to chloroplast imparted negative influence on the photosynthetic efficiency due to damaged thylakoids which resulted in lower chlorophyll contents [34]. Nevertheless, exogenous application of GA_3_ as foliar spray significantly buffered these negative effects in Cu-stressed plants. Previous literature also stated the protective role GA_3_ toward the photosynthetic machinery in metal contaminated soil [10]. The possible reason behind this mechanism might be the enhanced antioxidant activities that reduced the Cu-induced oxidative damages [35]. The decreased photosynthetic performances of plants without GA_3_ application may be linked to Cu toxicity and findings support the observation of Habiba et al. [36] who claimed that Cu toxicity caused a decline in chlorophyll biosynthesis and increased damage of thylakoid membranes. 

Copper stress rigorously impeded the performance of crop plants [37]. In addition, Cu stress promoted oxidative damage as inferred from the enhanced production of ROS. Increased synthesis of ROS and accumulation in plant tissues cause damage to cellular structures and macromolecules such as nucleic acid, proteins, lipids, and the photosynthetic apparatus [38]. However, it has been reported that increased synthesis of antioxidant enzymes such as SOD, POD, and CAT improved the response of *Oryza sativa* [39] and *Brassica napus* [40] plants under Cu stress. Accordingly, in the present study, the alleviation of oxidative stress resulted from enhanced antioxidant activities. The findings of our study also highlight the effectiveness of foliar applied GA_3_ in both Cu+ and Cu− soils. Foliar application of GA_3_ (100 mg·L^−1^) decreased the production of MDA, H_2_O_2_ contents, and electrolyte leakage compared to control (no GA_3_ application). In contrast, all concentrations of foliar applied GA_3_ enhanced the activities of SOD, POD, and CAT, but higher values for antioxidants enzymes were linked with 100 mg·L^−1^ GA_3_ (Figure 2 and Figure 3). The enhanced antioxidants activity can be considered as an indication of decreased accumulation of MDA and H_2_O_2_ contents [4,33,41]. These findings are supported by the results of Saleem et al. [4] who reported a similar effect of foliar applied GA_3_ to ameliorate the oxidative stress response in *Corchorus capsularis* L. Moreover, upregulation of activity of various antioxidant enzymes such as SOD, POD, and CAT shows the higher capacity of plants to scavenge excessive ROS under Cu stress [4]. The results of the current study are also in line with Fahad et al. [42], who argued that when the scavenging system against ROS is not effective, then crop plants become vulnerable to oxidative damages.

Depending on growth conditions, plants vary in their capacity of Cu uptake and accumulation. Plant roots play a central role in Cu uptake and transfer to stem and leaves through the xylem [43,44]. Pea plants treated with GA_3_ exhibited enhanced Cu accumulation in roots, leaves, and stem under Cu stress (Table 2). The increased gaseous exchange attributes such as photosynthesis and transpiration rate as the result of exogenous application of GA_3_ was likely the reason behind the increased Cu uptake by plants, even under phytotoxicity conditions [4,36]. The efficacy of GA_3_ to modulate the plant physiological status depends on its concentration, application method, and plant genetics [4]. The results of the present study also showed that the response of pea plants growth and development to GA_3_ application changed with change in concentration, mostly under Cu stressing conditions, overall, suggesting that GA_3_ promoted growth of pea plants and alleviated the Cu toxicity. To this end, foliar application of 100 mg·L^−1^ induced the best response in pea plants in terms morphological, physiological, and oxidative attributes. 

## 5. Conclusions

Gibberellic acid is a key regulator of several stages of plant growth, development, physiology, and morphology in plants, showing also pivotal regulatory effects against various environmental stressors. In this study, foliar application with different concentrations of GA_3_ proved to be successful for enhancing tolerance to Cu stress in pea plants, as evidenced from higher plant growth and antioxidant activity. In this view, the foliar application of a 100 mg·L^−1^ GA_3_ solution proved to be the most effective in enhancing tolerance to Cu and related oxidative stresses in pea plants, while at the same time maximizing the Cu content in plant organs. Results also reveal that GA3 alleviated Cu-induced stress on pea plants by stimulated activities of reactive oxygen-scavenging enzymes catalase, peroxidase, and superoxide dismutase, which not only helped in reducing electrolyte leakage, but also hindered accumulation of MDA and H_2_O_2_ in Cu-stressed pea plants. Overall, this indicates the possible role of this plant hormone in sustaining the phytoextraction functions of this important, N-fixing leguminous species, when crop rotations in Cu-polluted soils are concerned. 

## Figures and Tables

**Table 1:** Plant growth attributes of pea plants as affected by soil Cu contamination and GA_3_ foliar application.

Treatments		Plant Height (cm)	Plant Relative Fresh Weight (g)	Plant Dry Weight (g)	Leaves per Plant
Cu− soil	0 mg/L GA_3_	24.3 ± 0.33 ^e^	20.6 ± 0.38 ^ef^	9.0 ± 0.23 ^ef^	16.7 ± 0.38 ^e^
	10 mg/L GA_3_	27.3 ± 0.57 ^de^	22.1 ± 0.25 ^e^	11.3 ± 0.65 ^de^	18.3 ± 0.12 ^de^
	50 mg/L GA_3_	32.1 ± 1.15 ^bc^	28.1 ± 0.52 ^c^	14.4 ± 0.16 ^bc^	24.5 ± 0.54 ^bc^
	100 mg/L GA_3_	40.1 ± 1.13 ^a^	34 ± 1.2 ^a^	17.2 ± 0.12 ^a^	30.1 ± 0.82 ^a^
Cu+ soil	0 mg/L GA_3_	19.3 ± 0.88 ^f^	18.1 ± 0.57 ^f^	8.0 ± 0.54 ^f^	13.7 ± 0.23 ^e^
	10 mg/L GA_3_	23.3 ± 0.3 ^e^	20.3 ± 0.12 ^ef^	9.7 ± 0.43 ^def^	15.3 ± 0.13 ^e^
	50 mg/L GA_3_	28.7 ± 0.33 ^cd^	25.1 ± 0.92 ^d^	12.3 ± 0.27 ^cd^	21.7 ± 0.43 ^cd^
	100 mg/L GA_3_	34.2 ± 0.58 ^b^	31 ± 1.12 ^b^	15.2 ± 0.74 ^ab^	28.3 ± 0.73 ^ab^
HSD _(Interaction)_		3.773	2.614	2.669	4.76

Different letters indicate significant difference among treatment means according to Tukey’s honestly significant difference (HSD) test (*p* ≤ 0.05). Each value indicates mean of four replicates ± standard error (5 plants per replicate under bi-factorial CRD arrangement).

**Table 2:** Copper concentration in roots, stem, and leaves of peas as affected by exogenous application of GA_3_ and Cu contamination of soil.

Treatments		Root Cu (mg·kg^−1^)	Leaves Cu (mg·kg^−1^)	Stem Cu (mg·kg^−1^)
Cu− soil	0 mg/L GA_3_	16.7 ± 0.81 ^g^	31.7 ± 0.88 ^h^	22.1 ± 0.52 ^g^
	10 mg/L GA_3_	20.7 ± 0.78 ^g^	36.0 ± 1.15 ^g^	27.2 ± 0.57 ^fg^
	50 mg/L GA_3_	26.3 ± 0.88 ^f^	43.7 ± 1.76 ^f^	32.7 ± 0.72 ^f^
	100 mg/L GA_3_	34.7 ± 1.25 ^e^	58.1 ± 1.52 ^e^	39.7 ± 0.89 ^e^
Cu+ soil	0 mg/L GA_3_	61.3 ± 0.69 ^d^	145.2 ± 2.31 ^d^	82.7 ± 1.45 ^d^
	10 mg/L GA_3_	66.7 ± 0.89 ^c^	161.9 ± 1.15 ^c^	95.3 ± 0.88 ^c^
	50 mg/L GA_3_	74.3 ± 0.82 ^b^	177.3 ± 1.73 ^b^	119.9 ± 2.3 ^b^
	100 mg/L GA_3_	90.7 ± 1.2 ^a^	193.3 ± 1.85 ^a^	145.3 ± 2.33 ^a^
HSD _(Interaction)_		4.371	3.527	6.332

Different letters indicate significant difference among treatment means according to Tukey’s honestly significant difference (HSD) test (*p* ≤ 0.05). Each value indicates mean of four replicates ± standard error (10 plants per replicate under bi-factorial CRD arrangement).

**Table 3:** Correlation coefficients among variables and principal components factors.

Variables	Principle Component Factors	
	F1	F2
Plant height	0.992	−0.056
Plant relative fresh weight	0.989	0.082
Plant dry weight	0.997	0.025
Number of leaves	0.986	0.091
Chlorophyll content	0.974	−0.148
Photosynthetic rate	0.982	0.105
Transpiration rate	0.995	0.033
CO_2_ index	0.997	0.026
Malondialdehyde content	−0.983	0.056
H_2_O_2_ content	−0.979	−0.087
Electrolyte leakage	−0.997	−0.059
Superoxide dismutase	0.989	−0.094
Peroxidase	0.996	−0.022
Catalase	0.990	−0.098
Root Cu content	0.027	0.998
Leaves Cu content	−0.102	0.992
Stem Cu content	0.042	0.995
Eigenvalue	13.711	3.059
Explained variability (%)	80.652	17.997

## Data Availability

Data sharing not applicable.
